# ATRA-induced NEAT1 upregulation promotes autophagy during APL cell granulocytic differentiation

**DOI:** 10.1371/journal.pone.0316109

**Published:** 2024-12-23

**Authors:** Doudou Tang, Huihui Wang, Yafeng Jiang, Mingjie Chen, Guangsen Zhang, Shangjie Wu, Yewei Wang

**Affiliations:** 1 Department of Respiratory and Critical Care Medicine, the Second Xiangya Hospital, Central South University, Changsha, Hunan, China; 2 Hunan Centre for Evidence-based Medicine, Central South University, Changsha, Hunan, China; 3 Department of Hematology, the Second Xiangya Hospital, Central South University, Changsha, Hunan, China; 4 Institute of Molecular Hematology, Central South University, Changsha, Hunan, China; 5 Shanghai NewCore Biotechnology Co., Ltd., Minhang District, Shanghai, China; Florida Atlantic University, UNITED STATES OF AMERICA

## Abstract

**Aims:**

Acute promyelocytic leukemia (APL) progresses quickly and often leads to early hemorrhagic death. Treatment with *all-trans retinoic acid* (ATRA) promotes differentiation of APL cells and clinical remission, making APL a potentially curable malignancy. Understanding how ATRA works may lead to new treatments for other types of leukemia. Long non-coding RNA NEAT1 has been implicated in the differentiation of APL cells. This study aims to elucidate the specific role of NEAT1 in the granulocytic differentiation of APL.

**Methods:**

The influence of NEAT1 on autophagy and PML/RARα degradation was assessed using western blot assays. The impact of NEAT1 on the expression of autophagy-related genes was evaluated through quantitative real-time RT-PCR. Mechanistic insights into the role of NEAT1 in modulating autophagy were supported by RNA immunoprecipitation and RNA pulldown assays.

**Key findings:**

Knockdown of NEAT1 suppressed autophagy and attenuated ATRA-induced PML/RARα degradation and granulocytic differentiation of APL cells. Subsequent screening of autophagy-related genes demonstrated that silencing NEAT1 impaired the ATRA-induced upregulation of ATG10 and ATG12. Mechanistic investigations revealed that the RNA-binding protein TAF15 interacted with NEAT1, synergistically stabilizing the mRNA of ATG10 and ATG12. Furthermore, knockdown of NEAT1 impaired the interactions between TAF15 and the mRNAs of ATG10 and ATG12, thereby compromising their mRNA stability.

**Significance:**

Our study elucidates the critical role of NEAT1-mediated autophagy in the differentiation of APL cells and delineates the molecular mechanism by which upregulation of NEAT1 enhances autophagy. Specifically, NEAT1 binds to the RNA-binding protein TAF15, which in turn stabilizes the mRNA of both ATG10 and ATG12.

## Introduction

Acute promyelocytic leukemia (APL), a distinct subtype of acute myeloid leukemia (AML), is characterized by t(15;17) and the resulting promyelocytic leukemia/retinoic acid receptor α (PML/RARα) fusion gene. The hallmark of APL is the uncontrolled proliferation of leukemic blasts blocked at the promyelocyte stage of differentiation within bone marrow [1]. Accounting for 10–15% of all AML cases, APL is noted for its rapid progression and a high incidence of hemorrhagic death [2]. Treatment with pharmacological doses of *all-trans retinoic acid* (ATRA) can trigger terminal differentiation of APL blasts and disease remission, particularly when combined with arsenic trioxide or chemotherapy, establishing APL as the most curable subtype of AML [2, 3]. However, the efficacy of ATRA is primarily confined to APL [4]. Thus, advancing our understanding of ATRA’s mechanisms in inducing differentiation in APL is crucial for developing new treatments for other leukemia types. Previous studies had mainly focused on protein-coding genes in APL differentiation, leaving the roles of long non-coding RNAs (lncRNAs) in this process largely unexplored.

Macroautophagy (hereafter referred to as autophagy) is an evolutionarily conserved catabolic process that involves the formation of double-membrane autophagosomes. These autophagosomes engulf damaged organelles and protein aggregates for delivery to the lysosome, facilitating the rapid degradation of compromised cellular structures [5]. Autophagy-related genes (ATGs), which are also highly conserved, play critical roles in regulating autophagosome formation [6]. Although often categorized as a type of programmed cell death, autophagy primarily functions as a fundamental intracellular homeostatic mechanism and participates in a variety of physiological processes [7, 8]. For instance, autophagy has been shown to support the differentiation processes in hematopoiesis, including the formation of erythrocytes [9], lymphocytes [10], monocyte-macrophages [11] and plasma cells [12].

Recent studies have highlighted a significant role for autophagy in APL cell differentiation triggered by ATRA [13–15]. It is reported that ATRA enhanced autophagic activity in APL patient-derived NB4 cells. The inhibition of autophagy impaired ATRA-induced differentiation of NB4 cells, while the induction of autophagy through rapamycin not only caused differentiation of NB4 cells but also enhanced ATRA-mediated differentiation [13]. Further investigation demonstrated that inhibiting autophagy attenuated the degradation of PML-RARα oncoprotein, thereby impeding granulocytic differentiation. Conversely, rapamycin promoted ATRA-induced degradation of PML-RARα and enhanced differentiation [14]. These findings indicate that ATRA-induced autophagy is critical for PML-RARα degradation and the differentiation of APL cells. However, the specific molecular mechanisms by which ATRA induces autophagy in APL cells remain poorly elucidated.

A Recent study identified that lncRNA NEAT1 was repressed in APL and upregulated during ATRA-induced differentiation of APL cells, where silencing NEAT1 impaired ATRA-induced differentiation [16], indicating NEAT1’s involvement in APL cell differentiation. In this work, we found that knockdown of NEAT1 inhibited autophagy and attenuated ATRA-induced degradation of PML/RARα and differentiation in APL cells by impairing the upregulation of ATG10 and ATG12. Mechanistic investigations demonstrated that the RNA binding protein TAF15 interacted with NEAT1, synergistically stabilizing the mRNAs of ATG10 and ATG12, thereby promoting autophagy and facilitating granulocytic differentiation in APL cells. This study highlights the regulatory role of NEAT1 in autophagy and elucidates the molecular mechanism by which NEAT1 contributes to the differentiation of APL cells.

## Material and methods

### Cell culture and reagents

NB4 cells were maintained in RPMI 1640 medium (Gibco, Carlsbad, CA, USA) supplemented with 10% fetal bovine serum (FBS; Gibco). 293T cells were cultured in Dulbecco’s Modified Eagle Medium (DMEM; Gibco) with 10% FBS. Both cells were incubated in humidified atmosphere at 37ºC with 5% CO_2_. All-trans retinoic acid (ATRA; Sigma-Aldrich, St. Louis, MO, USA) was used at a final concentration of 1 μM. Polybrene and Actinomycin D were purchased from Sigma-Aldrich (St. Louis, MO, USA). Bafilomycin A1 (Sangon Biotech, Shanghai, China) were utilized at a final concentration of 25 nM.

### Quantitative real-time RT-PCR

Total RNA was extracted using the RNAiso Plus reagent (TaKaRa, Dalian, Liaoning, China). This RNA was subsequently reverse transcribed into cDNA using the PrimeScript RT Reagent Kit (TaKaRa). Quantitative real-time PCR (RT-qPCR) assays were conducted with the Roche LightCycler 96 system with SYBR Premix Ex Taq II (TaKaRa). GAPDH served as the normalization control. Relative gene expression at various time points was initially analyzed using the ΔCT method, which involves calculating the ratio of the target gene expression to that of GAPDH. Subsequently, relative expression levels were calculated and normalized to the control group (NC) baseline expression prior to ATRA treatment. Details of all primers used for RT-qPCR can be found in S1 Table.

### RNA interference experiment and transfection

Lentiviral plasmid constructs encoding short hairpin RNAs (shRNAs) targeting NEAT1 and TAF15, along with a negative control, were synthesized using the pLVX-shRNA2 vector (Clontech Laboratories, Mountain View, CA, USA) following the manufacturer’s protocol. Lentiviral particles were generated by co-transfecting 293T cells with the packaging plasmids pMD2.G and psPAX2. The viral supernatants were harvested 48 hours post-transfection and used to transduce NB4 cells in the presence of 8 μg/ml polybrene. Expression levels of the targeted genes were assessed via RT-qPCR.

The shRNA targeting NEAT1 was selected based on its documented efficacy in silencing NEAT1 across various cell types, as reported in prior studies [17–20], and its proven ability to reduce NEAT1 expression in NB4 cells [16]. Consequently, this specific shRNA sequence was utilized in our experiments. For TAF15 knockdown, a combination of three shRNAs from The RNAi Consortium (TRC) human genome-wide shRNA collection (TRCN0000020140, TRCN0000020141, and TRCN0000020143) was used, as detailed in the literature [21]. The sequence 5’-AGCGUGUAGCUAGCAGAGG-3’ served as the negative control.

### Flow cytometry

NB4 cells transfected with shRNA targeting NEAT1 (shNEAT1) or a negative control were collected following 48 hours of treatment with ATRA. Subsequently, these cells were stained using APC-conjugated antibodies against CD11b, CD11c, and CD18 (BioLegend, San Diego, CA, USA). Flow cytometric analysis was performed using a BD FACS Canto II system (BD Biosciences, San Jose, CA, USA) to assess the expression of these markers.

### Western blot

The detailed procedure was described previously [22]. Briefly, proteins extracted from cells were separated by SDS-polyacrylamide gel electrophoresis and subsequently transferred to PVDF membranes (Sigma). These membranes were then incubated with specific primary antibodies. Protein bands were visualized using an ECL detection kit (Invitrogen, Carlsbad, CA, USA). GAPDH served as the loading control. The primary antibodies used included anti-LC3B (Proteintech, 14600-1-AP), anti-p62 (Proteintech, 66184-1-Ig), anti-RARα (Santa Cruz Biotechnology, C-20x), anti-TAF15 (Abcam, ab134916), anti-ATG10 (Proteintech, 13406-1-AP), anti-ATG12 (Proteintech, 11264-1-AP), and anti-GAPDH (Proteintech, 10494-1-AP).

### Bioinformatics analysis

The correlations between NEAT1/TAF15 and ATG10/ATG12 in APL were analyzed using data from the Gene Expression Omnibus (GEO) datasets GSE10358 and GSE12662. Potential RNA-binding proteins that interact with NEAT1, ATG10, and ATG12 were predicted using the ENCORI platform (http://starbase.sysu.edu.cn/index.php). Additionally, the interaction scores between TAF15 and the mRNAs of ATG10, ATG12, and NEAT1 were predicted using the RNA-Protein Interaction Prediction (RPISeq) tool (http://pridb.gdcb.iastate.edu/RPISeq/).

### Subcellular fractionation location

Cytoplasmic and nuclear RNAs were separately isolated from NB4 cells and ATRA-treated NB4 cells following the instruction of PARIS kit (Invitrogen, Carlsbad, CA, USA). RT-qPCR was used to detect the RNA (GAPDH, U6, and NEAT1) levels in fractions.

### RNA immunoprecipitation assay

RNA immunoprecipitation (RIP) was performed by using the EZ-Magna RIP RNA-binding protein immunoprecipitation kit (Millipore, Billerica, MA, USA) according to the manufacturer’s instructions. Subsequently, the precipitated RNAs were subjected to RT-qPCR analysis. Fold enrichment was calculated based on CT as 2^-Δ(ΔCT)^, where ΔCt = CT_IP_- CT_Input_ and Δ(ΔCT) = ΔCT_antibody_ - ΔCT_IgG_.

### RNA pulldown assay

The detailed procedure was described previously [23]. In brief, the 3’-untranslated regions (3’-UTRs) of ATG10 and ATG12 were synthesized in vitro using T7 RNA polymerase (New England Biolabs, Ipswich, MA, USA). Subsequent purification was performed using the RNeasy Plus Mini Kit (Qiagen, Hilden, Germany), followed by treatment with RNase-free DNase I (Qiagen) to remove residual DNA. The purified transcripts were then biotin-labeled using the Biotin RNA Labeling Mix (Sigma-Aldrich). Protein lysates extracted from NB4 cells were incubated with the biotinylated 3’-UTRs of ATG10 and ATG12. Streptavidin-coated magnetic agarose beads were utilized to isolate the RNA-protein complexes. Finally, the complexes were analyzed via western blot assay to confirm the presence of specific proteins.

### Statistical analysis

The Student’s t test was used to assess the significance of differences in data obtained from at least three independent experiments. Data are presented as mean ± standard error of the mean (S.E.M.). P-values less than 0.05 were considered statistically significant. An asterisk (*) denotes *p* < 0.05.

## Results

### ATRA-induced NEAT1 upregulation contributes to autophagy and PML/RARα degradation during APL cell granulocytic differentiation

**Fig 1:**
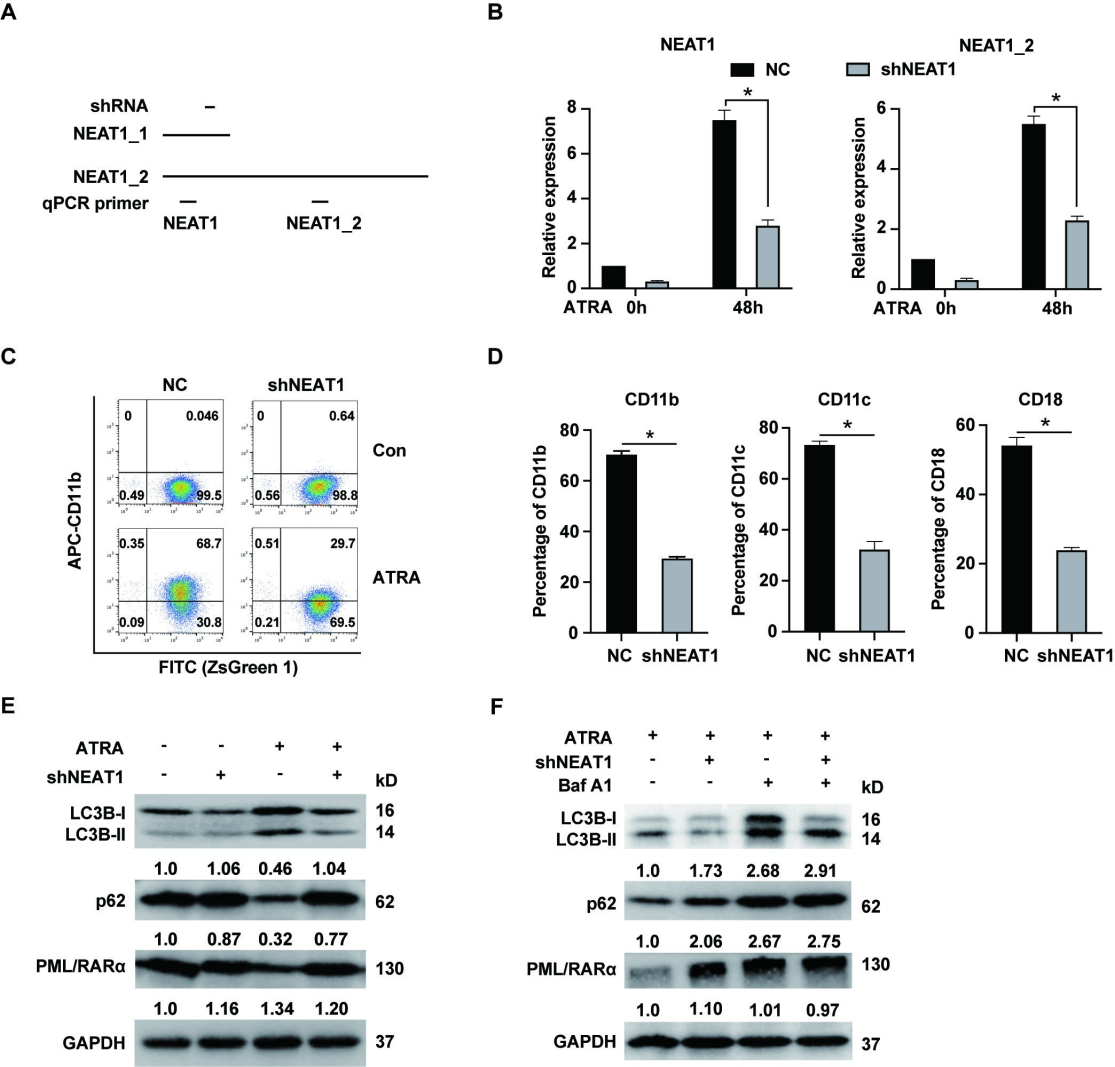
ATRA-induced upregulation of NEAT1 promotes autophagy and PML/RARα degradation during granulocytic differentiation of NB4 cells. (A) Schema of the two NEAT1 isoforms and the positions of qPCR primers and the shRNA targets. (B) NB4 cells were transfected with NEAT1-specific shRNA (shNEAT1) or negative control shRNA (NC). The expression levels of NEAT1 and its isoform NEAT1_2 were assessed in NB4 cells both before and after treatment with 1 μM ATRA for 48 hours by RT-qPCR. (C) The representative flow cytometry plots of NB4 cells stained with CD11b both before and after ATRA treatment. (D) The expression of granulocytic differentiation markers CD11b, CD11c and CD18 was determined in NB4 cells following treatment with 1 μM ATRA for 48 hours. (E) Protein levels of LC3B, p62 and PML/RARα were examined in NB4 cells both before and after 1 μM ATRA treatment for 48 hours. (F) The protein levels of LC3B, p62 and PML/RARα were also determined in NB4 cells treated with 1 μM ATRA treatment for 48 hours and 25 nM bafilomycin A1 for 12 hours. * indicates *p*<0.05.

It is reported that ATRA induced upregulation of NEAT1 in APL cells, and silencing NEAT1 impaired granulocytic differentiation in these cells [16]. However, the underlying mechanism by which NEAT1 influences APL cell differentiation remains unclear. It is known that NEAT1 exists in two isoforms, NEAT1_1 and NEAT1_2, as depicted in Fig 1A. The primer pair used for NEAT1 detection targets both isoforms, whereas the primer pair for NEAT1_2 is specific to this isoform. Here, we first constructed short hairpin RNA (shRNA) to stably silence NEAT1 in NB4 cells derived from APL patient. The shRNA design targeted a sequence common to both NEAT1_1 and NEAT1_2. Thus, we need to measure the expression level of NEAT1 and NEAT1_2. As shown in Fig 1B, shRNA effectively reduced the expression of NEAT1 and NEAT1_2 isoform in both untreated and ATRA-treated NB4 cells. Subsequently, we assessed the expression of granulocytic differentiation markers (CD11b, CD11c and CD18) in NB4 cells treated with ATRA for 48 hours, to confirm the role of NEAT1 in modulating differentiation in APL cells. Silencing NEAT1 significantly attenuated the ATRA-induced upregulation of CD11b, CD11c, and CD18 in APL cells, as revealed in Figs 1C, 1D and S1. This suggested that ATRA-induced NEAT1 upregulation played a crucial role in promoting granulocytic differentiation in these cells. Given that autophagy has been implicated in the granulocytic differentiation of APL cells and that the degradation of the PML/RARα oncoprotein by ATRA is autophagy-dependent [13], we investigated the protein levels of LC3B-II, p62 and PML/RARα in NEAT1-silenced NB4 cells, both before and after ATRA treatment. As shown in Fig 1E, ATRA treatment increased LC3B-II level, but this upregulation was impaired by NEAT1 knockdown, indicating that NEAT1 is essential for the formation of autophagosomes during ATRA treatment. Furthermore, ATRA treatment typically reduces p62 level, consistent with previous findings [14]. However, in NEAT1-knockdown cells, there was an accumulation of p62, suggesting an interruption in autophagic flux. Additionally, while ATRA normally promotes the degradation of the PML/RARα protein, this effect was reversed in the context of NEAT1 silencing. We further explored the autophagic flux by treating NB4 cells with both ATRA and bafilomycin A1, an autophagy inhibitor, as shown in Fig 1F. This treatment led to increased levels of LC3B-II and p62, indicating an accumulation of autophagic substrates due to inhibited degradation. Similarly, NEAT1 knockdown reduced LC3B-II accumulation, and bafilomycin A1 treatment diminished the ATRA-induced degradation of PML/RARα. These results collectively demonstrated that NEAT1 upregulation during APL cell differentiation facilitated autophagic processes and contributed to the degradation of the PML/RARα oncoprotein.

### Knockdown of NEAT1 reduced ATG10 and ATG12 upregulation induced by ATRA

**Fig 2:**
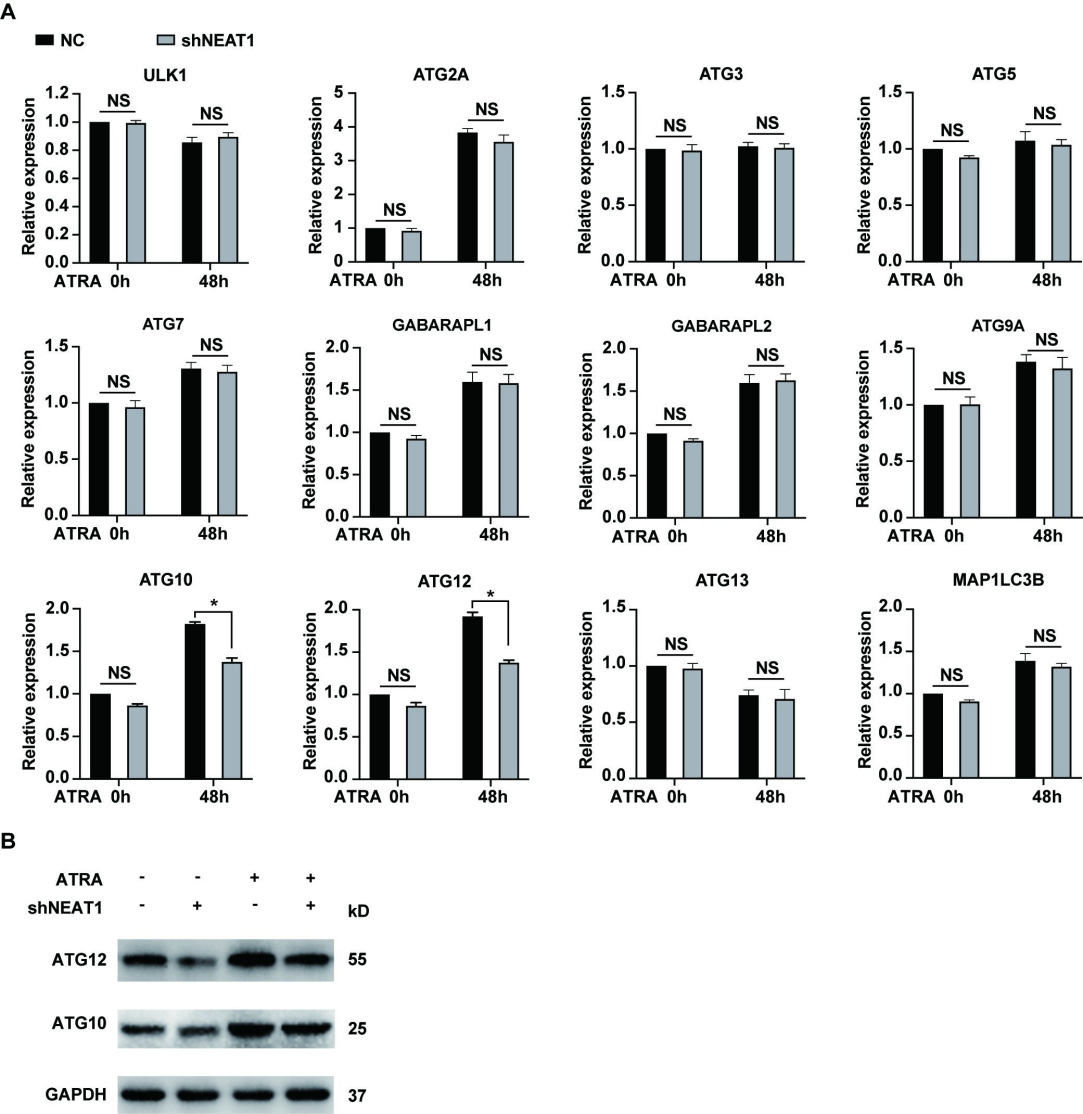
Knockdown of NEAT1 attenuates the ATRA-induced upregulation of ATG10 and ATG12. (A) Expression levels of the 12 autophagy-related genes were assessed in NB4 cells transfected with shNEAT1 and negative control shRNA (NC) both before and after treatment with 1 μM ATRA for 48 hours. * indicates *p*<0.05. “NS” indicates findings that are not statistically significant. (B) Protein levels of ATG10 and ATG12 were also tested in NB4 cells following treatment with 1 μM ATRA for 48 hours.

Because blocking Beclin 1 does not influence ATRA-induced granulocytic differentiation [15], we assessed the expression levels of 12 autophagy-related genes (ATGs) in NEAT1-knockdown NB4 cells before and after ATRA treatment. These ATGs are well-established and critical regulators of autophagy [7]. For instance, ULK1 is essential for the initiation of autophagy. Additionally, two protein conjugation systems, composed of ATG12/ATG7/ATG10/ATG5 and LC3/ATG3, are integral for autophagosome elongation and maturation. As shown in Fig 2, the upregulation of ATG10 and ATG12 were attenuated following NEAT1 knockdown, whereas the expression levels of other ATGs remained largely unaffected by the silencing of NEAT1. Furthermore, an analysis of the GEO datasets (GSE10358 and GSE12662) revealed significant positive correlations between NEAT1 and both ATG10 and ATG12 in APL (S2 Fig). These data support the role of NEAT1 in regulating the mRNA levels of ATG10 and ATG12. Collectively, these findings suggested that silencing NEAT1 decreased ATRA-induced upregulation of ATG10 and ATG12, thereby reducing autophagy and consequently the degradation of the PML/RARα oncoprotein.

### RNA binding protein TAF15 binds to and stabilizes ATG10 and ATG12 mRNA during APL cell granulocytic differentiation

**Fig 3:**
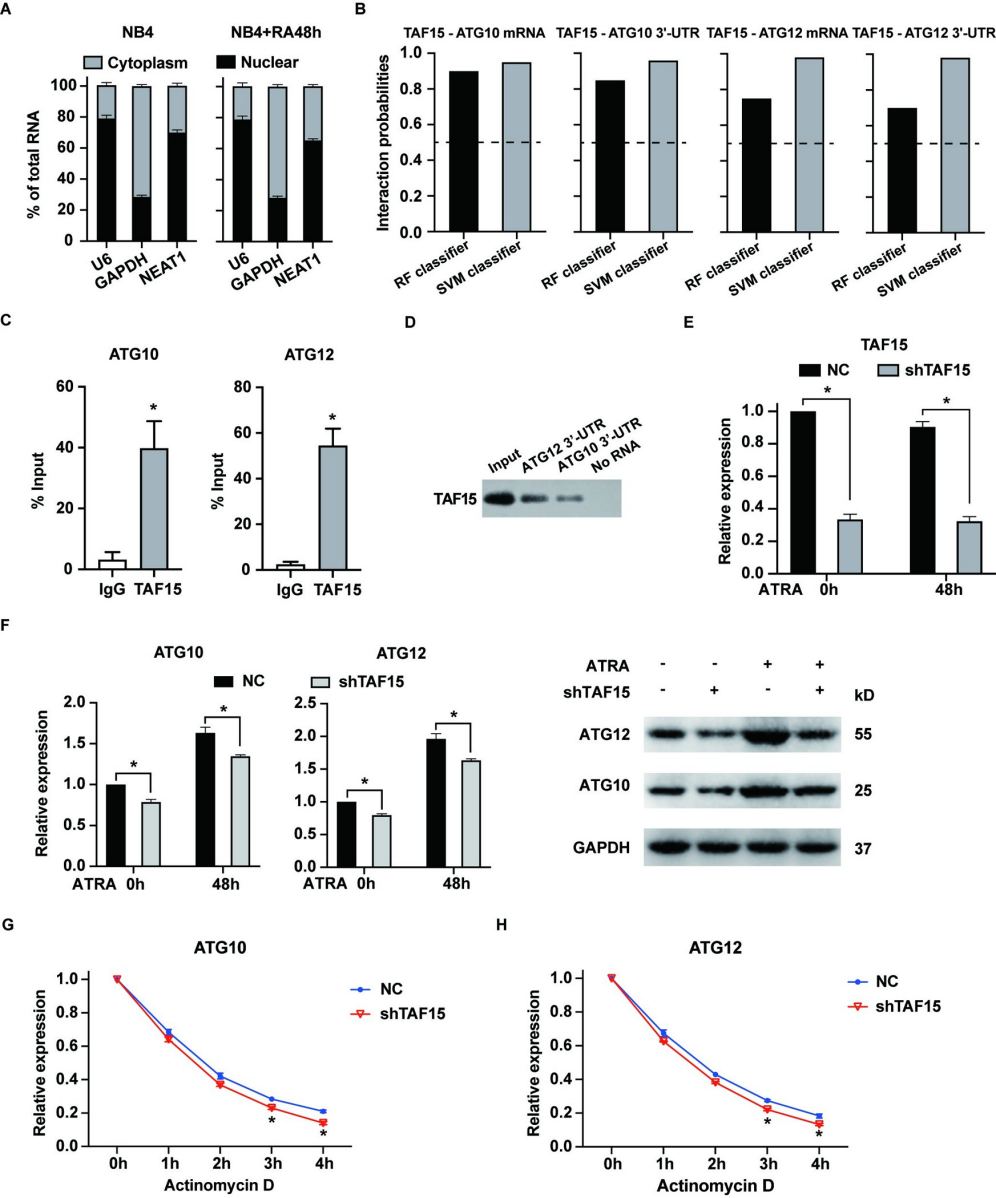
TAF15 binds to and stabilizes ATG10 and ATG12 mRNA in NB4 cells. (A) The expression of NEAT1 in the cytoplasm and nucleus of NB4 cells, both untreated and treated with ATRA, was determined by RT-qPCR. GAPDH served as a marker for the cytoplasm, and U6 as a marker for the nucleus. (B) The likelihood of TAF15 binding to the mRNA/3’-UTR regions of ATG10 and ATG12 were predicted by the RNA-Protein interaction prediction (RPISeq) website. *“*RF classifier” means random forest classifier and “SVM classifier” means support vector machine classifier. A prediction probability >0.5 was considered “positive”, indicating a probable interaction between the RNA and protein. (C) RIP experiments were conducted in ATRA-treated NB4 cells to confirm the interaction between TAF15 and the mRNA of ATG10 and ATG12. (D) Biotinylated probes of the 3’-UTR regions of ATG10 and ATG12 were employed to pull down TAF15 from cell lysates. The presence of TAF15 in the precipitates was detected using western blot assay. (E) NB4 cells were transfected with shRNA specifically targeting TAF15 (shTAF15) or negative control shRNA (NC). TAF15 expression was detected by RT-qPCR in these cells both before and after treatment with 1 μM ATRA for 48 hours. (F) The RNA and protein levels of ATG10 and ATG12 were measured in TAF15-silenced NB4 cells before and after ATRA treatment for 48 hours. (G-H) NB4 cells with or without TAF15 knockdown were treated with 1 μM ATRA for 48 hours, then the cells were further treated with Actinomycin D to inhibit new RNA synthesis. The expression levels of ATG10 and ATG12 mRNA were determined every hour by RT-qPCR. The data represent the mean ± S.E.M. from three replicates. * indicates *p*<0.05.

To investigate the potential molecular mechanism by which NEAT1 regulates ATG10 and ATG12, we analyzed the subcellular localization of NEAT1 in NB4 cells, both untreated and treated with ATRA. Consistent with its known role as a crucial component of paraspeckles, NEAT1 was predominantly localized in the nucleus (Fig 3A) [24]. However, a significant fraction, over 30%, of NEAT1 was also detected in the cytoplasm, aligning with previous studies that have documented its presence in both nuclear and cytoplasmic compartments [25]. NEAT1 is implicated in promoting cancer cell proliferation and autophagy through a competing endogenous RNA (ceRNA) mechanism, highlighting its functional importance in the cytoplasm as well [26, 27]. Cytoplasmic lncRNAs are known to interact with RNA binding proteins (RBPs) [28], and NEAT1 has been identified to interact with several RBPs, such as AUF1 [29], LIN28B [30], U2AF2 [31] and HuR [32]. Analysis using the ENCORI database revealed potential overlaps in the RBPs interacting with NEAT1, ATG10, and ATG12, identifying six RBPs ‐ ELAVL1 (HuR), U2AF2, TAF15, DDX54, HNRNPC, and RBFOX2 (S2–S4 Tables). Of which, ELAVL1 and U2AF2 are already known to interact with NEAT1 [31, 32]. DDX54 is associated with alterations in RNA secondary structure, HNRNPC is involved in pre-mRNA processing and mRNA metabolism, and RBFOX2 regulates alternative splicing in the nervous system. All these three RBPs are generally not linked directly to lncRNA stability. Conversely, TAF15, which acts as an mRNA stabilizer [33] and has not yet been reported to interact with NEAT1, ATG10 or ATG12, presents a novel candidate for further exploration of its potential interactions with NEAT1.

First, we hypothesized that TAF15 might stabilize ATG10 and ATG12 mRNA in APL cells. Notably, a strong correlation was observed between TAF15 and these mRNAs in APL, as shown in S3 Fig. We utilized the RPISeq website to predict the interaction probabilities between TAF15 and ATG10/ATG12 mRNA, which indicated high likelihoods of interaction (Fig 3B). Subsequently, the interactions between TAF15 and these mRNAs were experimentally confirmed using RNA immunoprecipitation (RIP) and RNA pulldown assays (Fig 3C and 3D). To further investigate the role of TAF15, we employed shRNA to specifically knock down TAF15 expression in NB4 cells (Fig 3E). As expected, TAF15 knockdown significantly reduced the expression levels of ATG10 and ATG12, both in untreated and ATRA-treated NB4 cells (Fig 3F). Finally, we assessed the impact of TAF15 silencing on the stability of ATG10 and ATG12 mRNA. The results demonstrated that TAF15 knockdown significantly compromised the mRNA stability of both ATG10 and ATG12 (Fig 3G and 3H). Collectively, these findings support the conclusion that TAF15 directly interacts with and stabilizes ATG10 and ATG12 mRNA, contributing to granulocytic differentiation in APL cells.

### NEAT1 and TAF15 synergistically stabilize ATG10 and ATG12 mRNA

**Fig 4:**
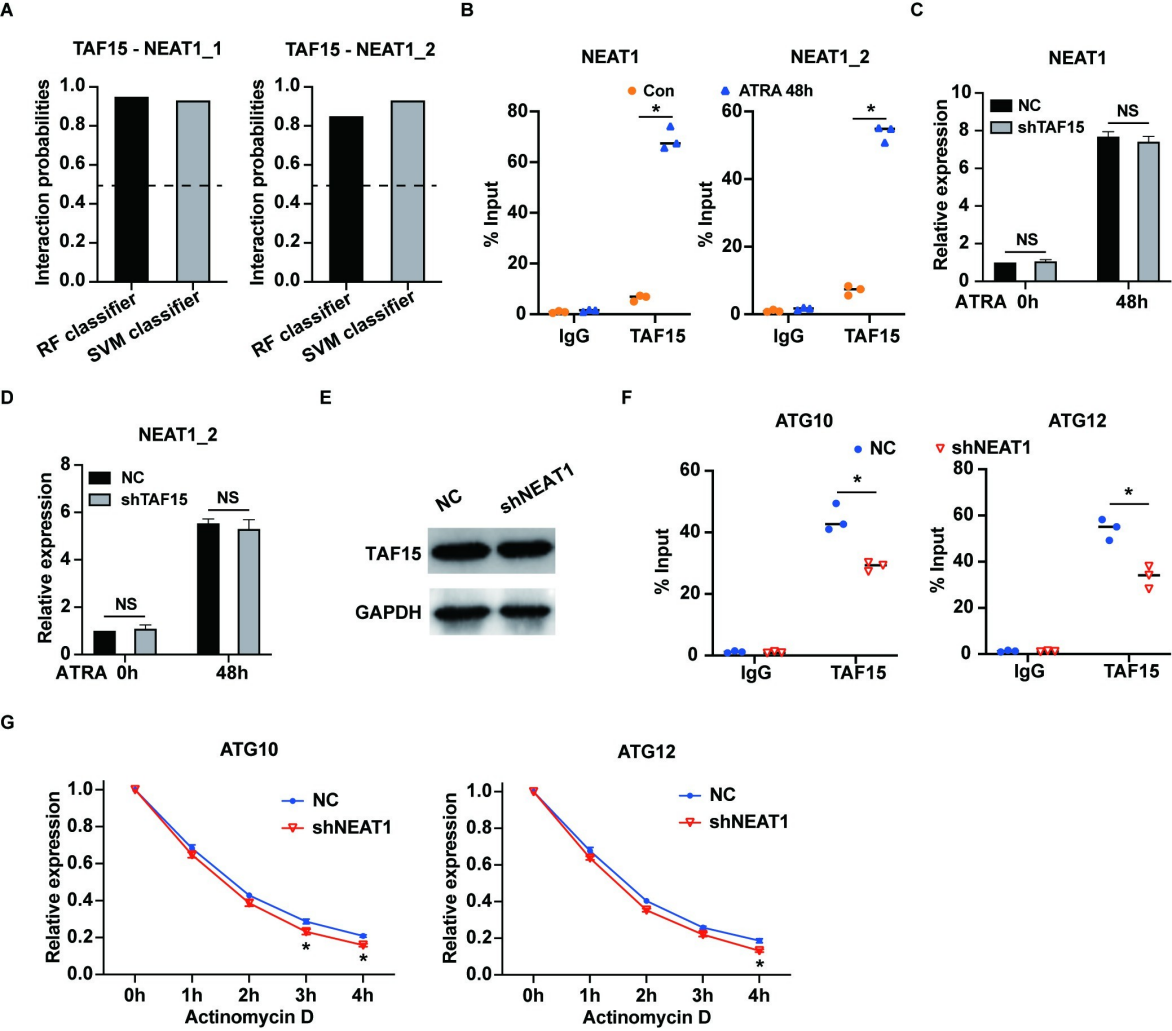
NEAT1 and TAF15 synergistically stabilize ATG10 and ATG12 mRNA. (A) The interaction probabilities between TAF15 and the two isoforms of NEAT1, NEAT1_1 and NEAT1_2 were predicted by the RPISeq website. (B) RIP assays were performed in NB4 cells and ATRA-treated NB4 cells to verify the interactions between TAF15 and NEAT1/NEAT1_2. (C) and (D) Expression levels of NEAT1 and NEAT1_2 isoform were examined in NB4 cells both with or without TAF15 knockdown before and after 1 μM ATRA treatment for 48 hours. (E) The protein level of TAF15 was determined in NB4 cells transfected with shNEAT1 and negative control (NC) after ATRA treatment for 48 hours. (F) RIP experiments were performed in NB4 cells transfected with shNEAT1 or negative control (NC) after ATRA treatment for 48 hours. The results indicated that the interactions between TAF15 and the mRNAs of ATG10 and ATG12 were reduced after NEAT1 knockdown. (G) NB4 cells transfected with shNEAT1 or negative control (NC) were treated with 1 μM ATRA for 48 hours, and subsequently the cells were further treated with Actinomycin D to inhibit new RNA synthesis. The expression levels of ATG10 and ATG12 mRNA were measured in NB4 cells every hour by RT-qPCR. The data represent the mean ± S.E.M. from three independent experiments. * indicates p<0.05. NS: not significant.

Furthermore, we utilized the RPISeq website to predict the interaction between TAF15 and the NEAT1 isoforms NEAT1_1 and NEAT1_2. The predictions indicated a probable interaction between TAF15 and both isoforms of NEAT1 (Fig 4A). These predictions were subsequently confirmed by RNA immunoprecipitation (RIP) assays conducted on ATRA-treated NB4 cells (Fig 4B). We also evaluated the expression levels of NEAT1 and its NEAT1_2 isoform in NB4 cells following TAF15 knockdown, both before and after ATRA treatment. Interestingly, TAF15 silencing had minimal impact on the expression levels of NEAT1 and its isoforms (Fig 4C and 4D). Similarly, inhibiting NEAT1 did not affect TAF15 expression (Fig 4E).

We further examined the interactions between TAF15 and ATG10/ATG12 mRNA in NB4 cells with NEAT1 knockdown following ATRA treatment. The results revealed that silencing NEAT1 significantly impaired the interactions between TAF15 and both ATG10 and ATG12 mRNAs (Fig 4F). Consistently, NEAT1 knockdown significantly reduced the stability of ATG10 and ATG12 mRNA (Fig 4G). Collectively, our findings suggested that NEAT1 and TAF15 work synergistically to stabilize ATG10 and ATG12 mRNA, thereby facilitating granulocytic differentiation in APL cells.

## Discussion

LncRNA NEAT1 has been implicated in various pathophysiological processes by modulating autophagy. For instance, NEAT1 upregulates ATG3 to enhance autophagy, thereby increasing resistance to sorafenib in hepatocellular carcinoma cells [34]. Conversely, silencing NEAT1 reduces autophagy by influencing ATG9A and ATG4B, which increases sensitivity to 5-fluorouracil in colorectal cancer [27]. Additionally, NEAT1 upregulates ATG9A, contributing to IGFBPrP1-induced autophagy and activation in hepatic stellate cells during liver fibrosis [35]. In contrast, NEAT1 could mitigate LPS‑induced inflammation by activating autophagy [36]. Furthermore, NEAT1 is associated with the modulation of autophagy in diseases such as congenital heart disease [37], Parkinson’s disease [38] and myocardial ischemia-reperfusion injury [39]. In our study, we have discovered that upregulation of NEAT1 enhances autophagy, which in turn promotes the degradation of PML-RARα and granulocytic differentiation of APL cells. This finding represents the first demonstration of NEAT1’s role in ATRA-induced autophagy and its contribution to APL cell differentiation via autophagy regulation. Consequently, enhancing NEAT1 expression may improve the efficacy of ATRA treatment, positioning NEAT1 as a potential therapeutic target for acute promyelocytic leukemia.

NEAT1 is known to interact with RNA binding proteins such as NONO and PSF, enhancing the processing of primary microRNAs globally [40]. Additionally, NEAT1 physically interacts with FUS, promoting cell growth in breast cancer [41]. When bound and stabilized by HuR, NEAT1 facilitates the proliferation and invasion of ovarian cancer cells [32]. Moreover, NEAT1 recruits EZH2 to gene promoters, thereby supporting myoblast proliferation during myogenesis [42]. These findings suggest that NEAT1 could interact with RBPs to fulfill diverse biological roles. On the other hand, RNA binding protein TAF15, acting as an RNA stabilizer, is reported to be recruited by lncRNA PITPNA‐AS1 to stabilize HMGB3 mRNA, enhancing proliferation and migration of lung squamous cell carcinoma cells [33]. Similarly, TAF15 interacts with TRPM2-AS to maintain the stability of TRPM2 mRNA, promoting cell proliferation in colorectal cancer [43]. These observations suggest that TAF15 might interact with other lncRNAs, including NEAT1. In this study, by predicting with the ENCORI database, TAF15 was identified as a shared RBP for NEAT1, ATG10 and ATG12 mRNA. Experimental validations confirmed that TAF15 interacts with NEAT1, synergistically stabilizing ATG10 and ATG12 mRNA. This research highlights, for the first time, the interaction between NEAT1 and RBP TAF15, which stabilizes downstream effectors. However, silencing TAF15 had minimal impact on NEAT1 expression, and blocking NEAT1 did not affect TAF15 expression. Given NEAT1’s role in structuring paraspeckles and its ability to scaffold RBPs [40], it is plausible that NEAT1 serves as a platform to recruit TAF15, thereby stabilizing downstream mRNAs such as ATG10 and ATG12.

Autophagy is characterized by the formation of autophagosomes, a process that involves several sequential stages: initiation, nucleation, elongation, maturation, and degradation. Two protein conjugation systems, composed of ATG12/ATG7/ATG10/ATG5 and LC3/ATG3, which are respectively triggered by ubiquitin-like molecules ATG12 and LC3, are required to autophagosome elongation and maturation [44]. Initially, ATG12 is activated by ATG7, transferred to ATG10, and then conjugated to ATG5 [45, 46]. The ATG12-ATG5 complex exhibits E3-like activity, facilitating the lipidation of phosphatidylethanolamine to LC3, thereby promoting the conversion of LC3-I into LC3-II [47], ATG12 is central to autophagy, with its knockdown disrupting autophagosome formation. ATG10, functioning as an E2-like conjugating enzyme, plays a crucial role in recruiting molecules for complex conjugation and is vital for autophagosome formation [48]. HuR has been shown to enhance the translation of ATG12 mRNA by binding to its 3’-UTR, thus facilitating autophagosome formation in hepatocellular carcinoma cells [49]. Additionally, CELF2 has been reported to increase ATG12 levels by modulating mRNA stability, thereby enhancing autophagic flux in colorectal cancer [50]. Furthermore, PTPB1 directly interacts with ATG10 mRNA and negatively regulates its expression, promoting tumor metastasis in colorectal cancer cells [51]. These findings highlight how upstream regulators influence autophagy by interacting with mRNAs of ATG12 and ATG10. In our study, we demonstrated that NEAT1 and TAF15 synergistically stabilized the mRNA of ATG10 and ATG12, enhancing autophagy during granulocytic differentiation of APL cells. Whereas knocking down NEAT1 or TAF15 reduces both the expression level and stability of ATG10 and ATG12 mRNA. Given that ATG10 and ATG12 are upstream regulators of LC3 and play a core role in autophagosome maturation, knockdown of TAF15 reduced the levels of ATG10 and ATG12, so silencing TAF15 would result in a reduction in LC3B-II levels and impair autophagic activity. Additionally, the combination of shNEAT1 and bafilomycin A1 appeared less effective than bafilomycin A1 alone in mitigating the effects of ATRA, which may be attributed to distinct mechanisms of autophagy regulation.

A key feature of the ATRA response in APL is the degradation of the PML-RARα oncoprotein, which facilitates cell differentiation and contributes to clinical remission. Besides autophagy, the proteasome and caspase pathways are also implicated in the degradation of PML-RARα [52–54]. The involvement of multiple pathways may explain why NEAT1 knockdown does not completely prevent the ATRA-induced degradation of PML-RARα. Additionally, the inability to fully reverse PML-RARα degradation could be attributed to the incomplete silencing of NEAT1 by shRNA.

A limitation of our study is that the findings were solely demonstrated using the NB4 cell line, derived from an APL patient, without validation in patient samples or mouse models. Collecting samples from APL patients is challenging due to the rarity of this subtype of acute myeloid leukemia. Additionally, the limited number of cells typically obtained from clinical samples and the significant variation between individual cases complicate the execution of mechanistic studies using patient-derived materials. Furthermore, there is currently no established appropriate mouse model for APL. Consequently, similar to many mechanistic studies, we relied on a cell line model to conduct our experiments. Moreover, due to the large size of NEAT1_2 (23 kb), constructing an overexpression plasmid is challenging, which has prevented us from conducting overexpression or inducible NEAT1 experiments.

**Fig 5:**
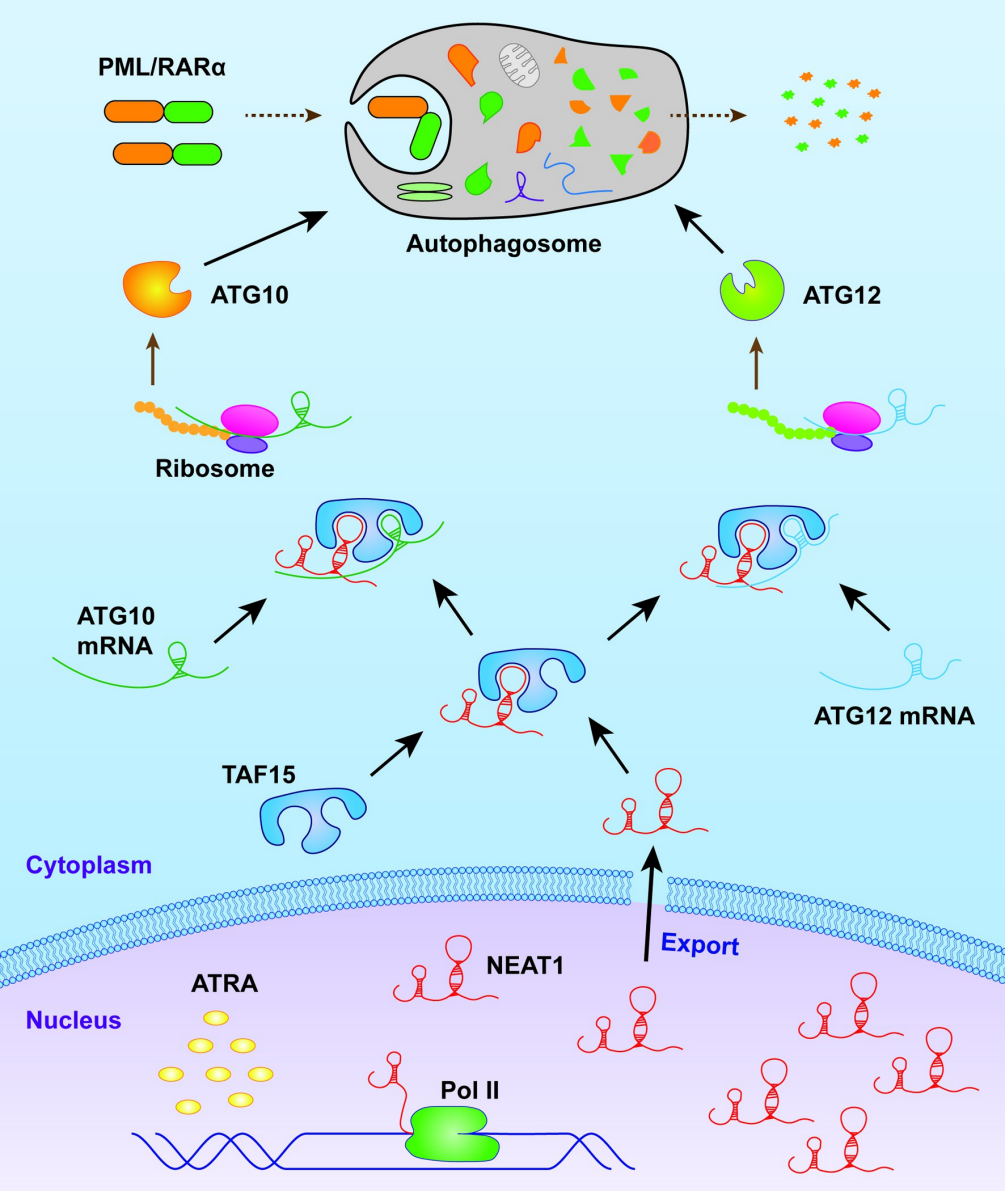
Schematic diagram illustrating the role of NEAT1 in enhancing autophagy during granulocytic differentiation in APL cells.

In summary, our results not only highlight the crucial role of NEAT1-mediated autophagy in the granulocytic differentiation of APL cells but also elucidate the molecular mechanism by which NEAT1 upregulation enhances autophagy. Specifically, NEAT1 interacts with TAF15 to stabilize the mRNAs of ATG10 and ATG12, thereby promoting autophagy (Fig 5). This study underscores a novel regulatory pathway that may be targeted therapeutically to modulate differentiation in APL.

## Supporting information

S1 Fig The representative flow cytometry plots of NB4 cells stained with CD11c and CD18 both before and after ATRA treatment. (TIF)

S2 Fig The correlations between NEAT1 and ATG10 and ATG12 in APL were analyzed using data retrieved from the GEO datasets GSE10358 and GSE12662. (TIF)

S3 Fig The correlations between TAF15 and ATG10 and ATG12 in APL were analyzed using data retrieved from the GEO datasets GSE10358 and GSE12662. (TIF)

S1 Table Primers used for RT-qPCR. (XLSX)

S2 Table ENCORI predicted NEAT1 interacted RNA binding proteins. (XLSX)

S3 Table ENCORI predicted ATG10 interacted RNA binding proteins. (XLSX)

S4 Table ENCORI predicted ATG12 interacted RNA binding proteins. (XLSX)

S1 File Original western blot figures. (PPTX)
