# Supplementary figures and images for: ATRA-induced NEAT1 upregulation promotes autophagy during APL cell granulocytic differentiation
Source: PLoS One. 2024 Dec 23;19(12):e0316109. doi: 10.1371/journal.pone.0316109 (PMC11666005; doi:10.1371/journal.pone.0316109)

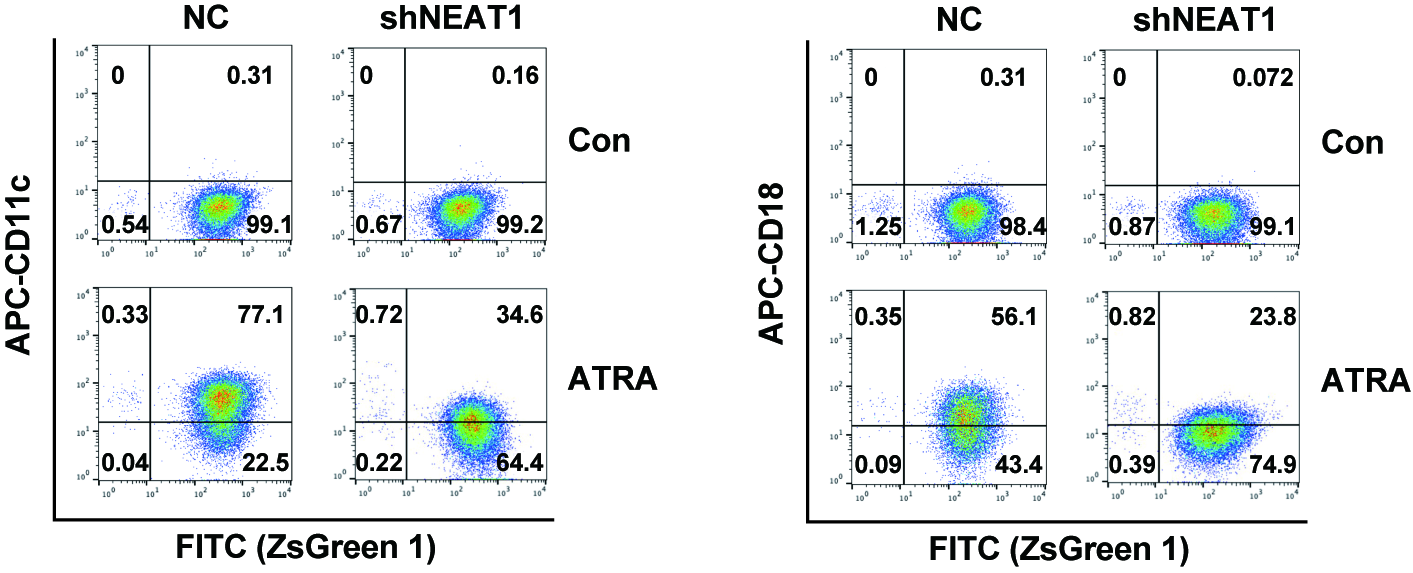

Supplement: S1 Fig — (TIF) [file pone.0316109.s001.tif]

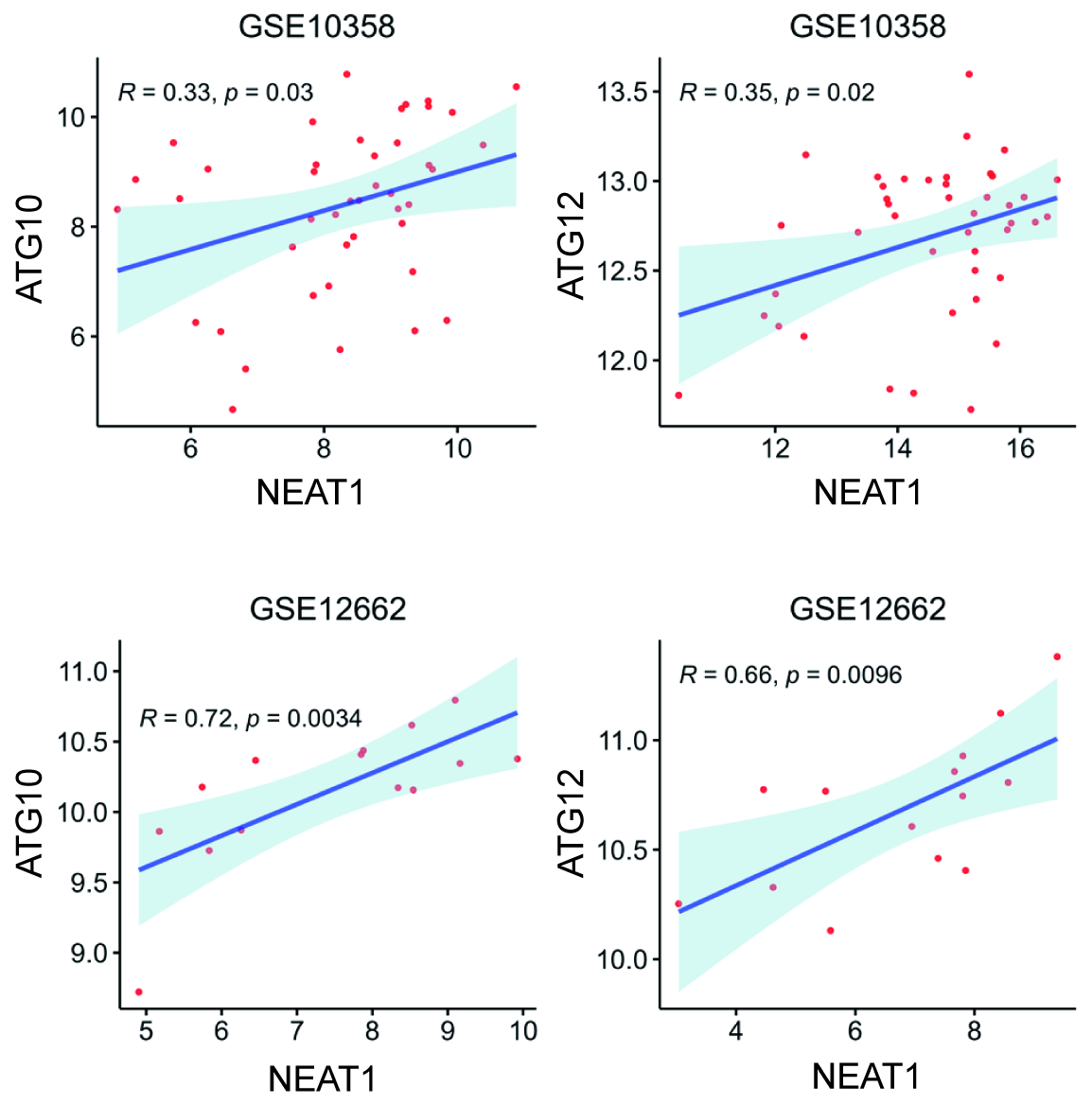

Supplement: S2 Fig — (TIF) [file pone.0316109.s002.tif]

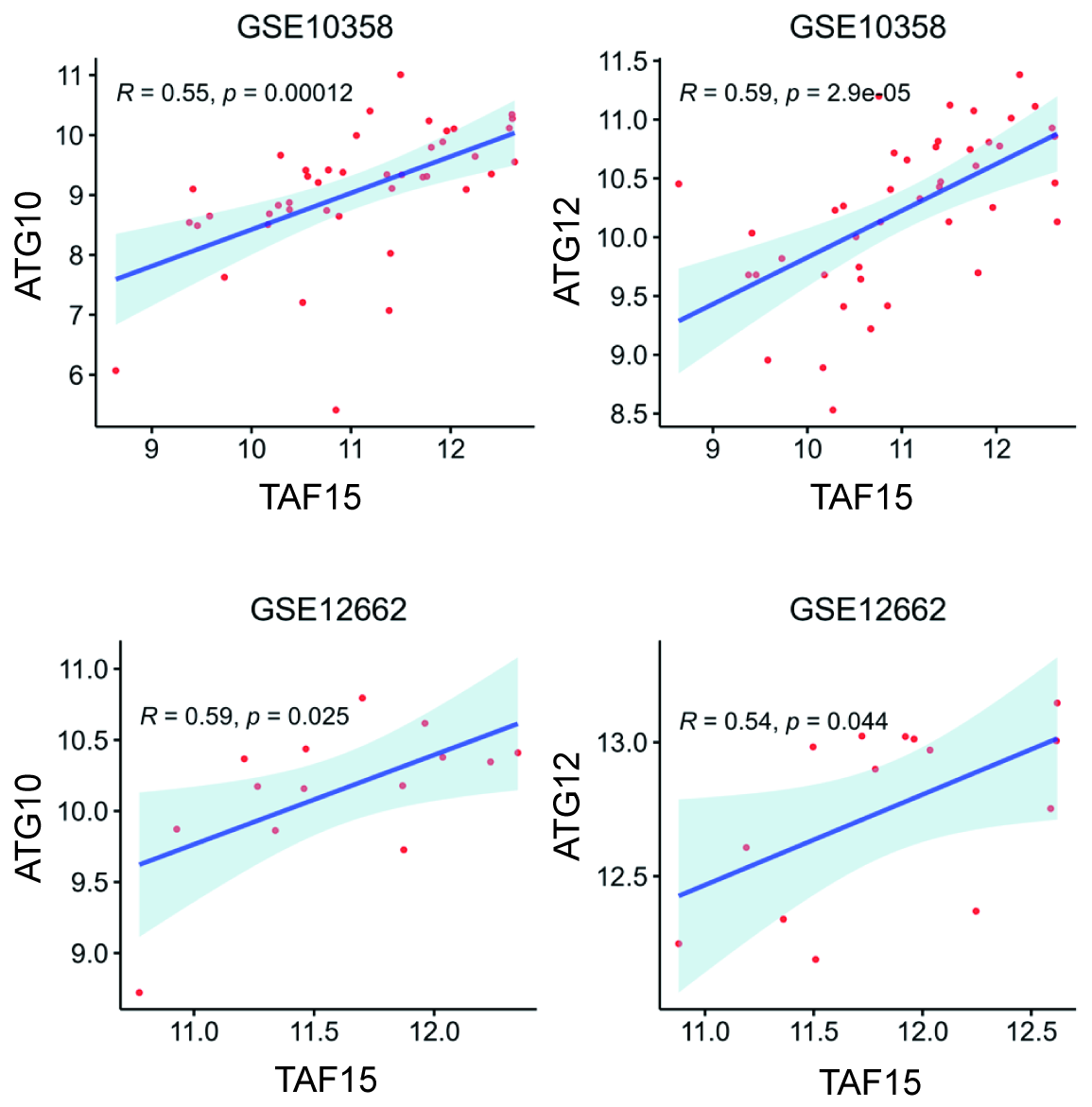

Supplement: S3 Fig — (TIF) [file pone.0316109.s003.tif]
