# Supplementary material for: ATRA-induced NEAT1 upregulation promotes autophagy during APL cell granulocytic differentiation
Source: PLoS One. 2024 Dec 23;19(12):e0316109. doi: 10.1371/journal.pone.0316109 (PMC11666005; doi:10.1371/journal.pone.0316109)
Supplement: S1 File — (PPTX) [file pone.0316109.s008.pptx]

## Slide 1
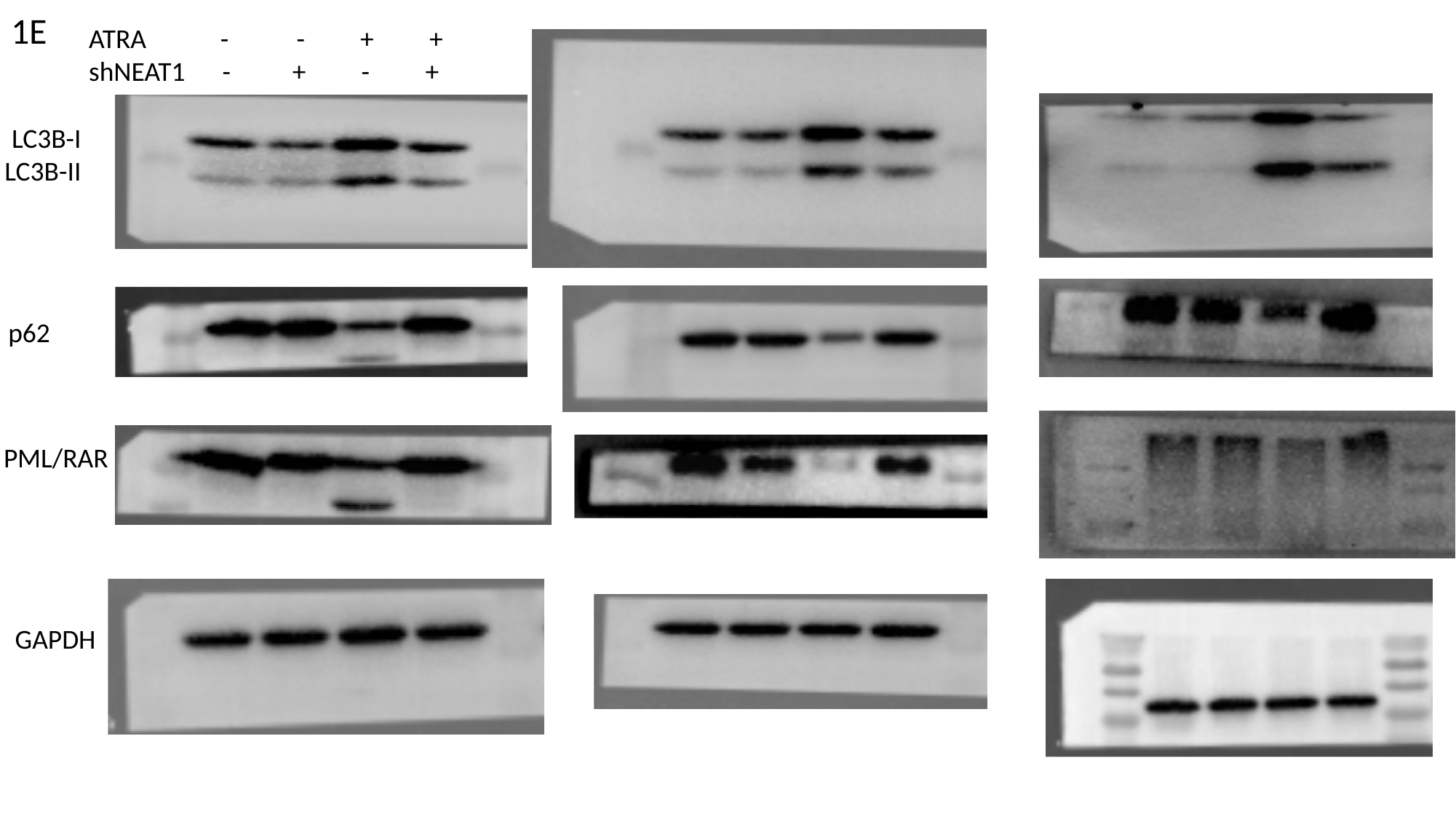

1E
ATRA - - + +
shNEAT1 - + - +
LC3B-I
LC3B-II
p62
PML/RAR
GAPDH

## Slide 2
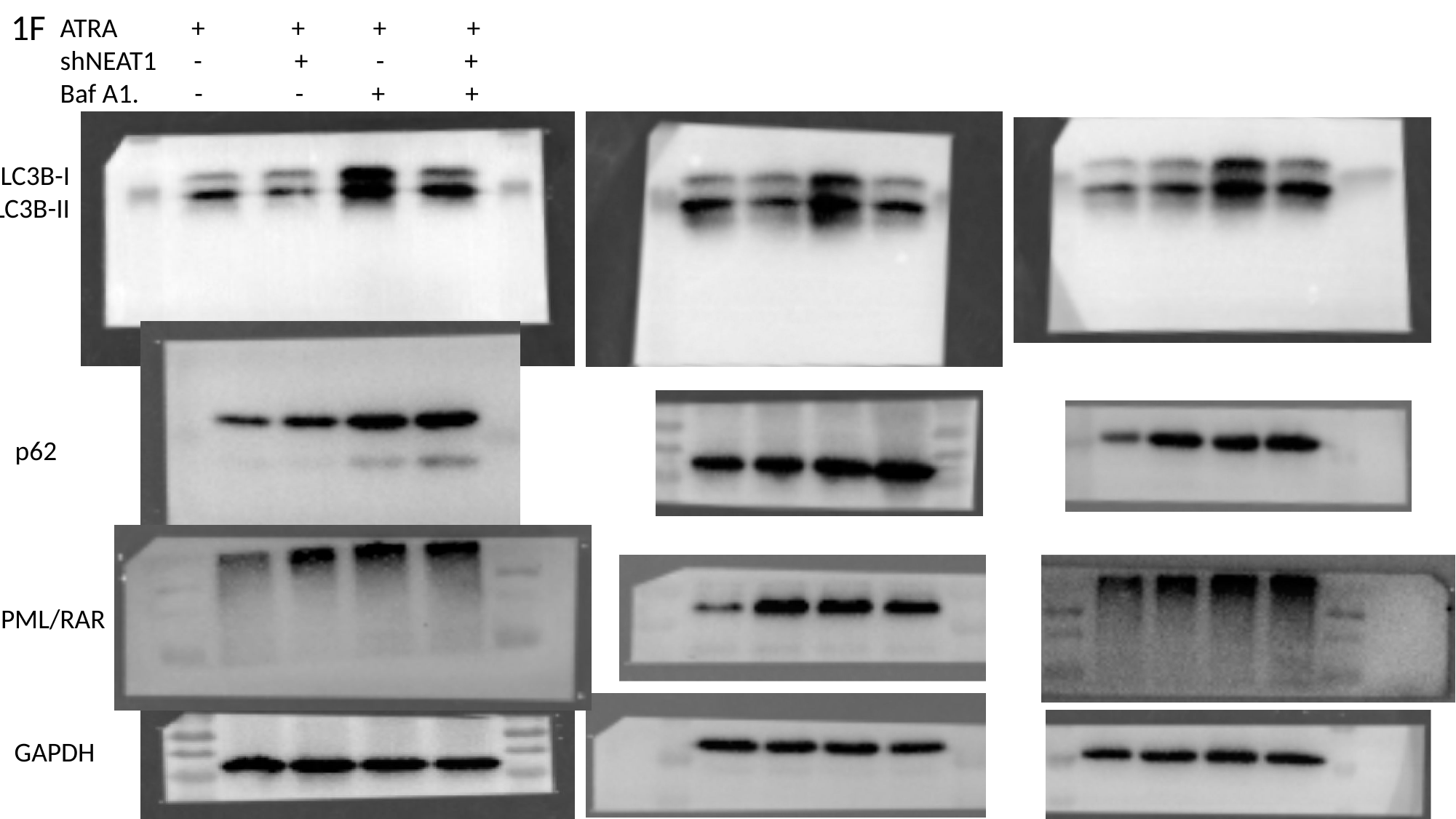

1F
ATRA + + + +
shNEAT1 - + - +
Baf A1. - - + +
LC3B-I
LC3B-II
p62
PML/RAR
GAPDH

## Slide 3
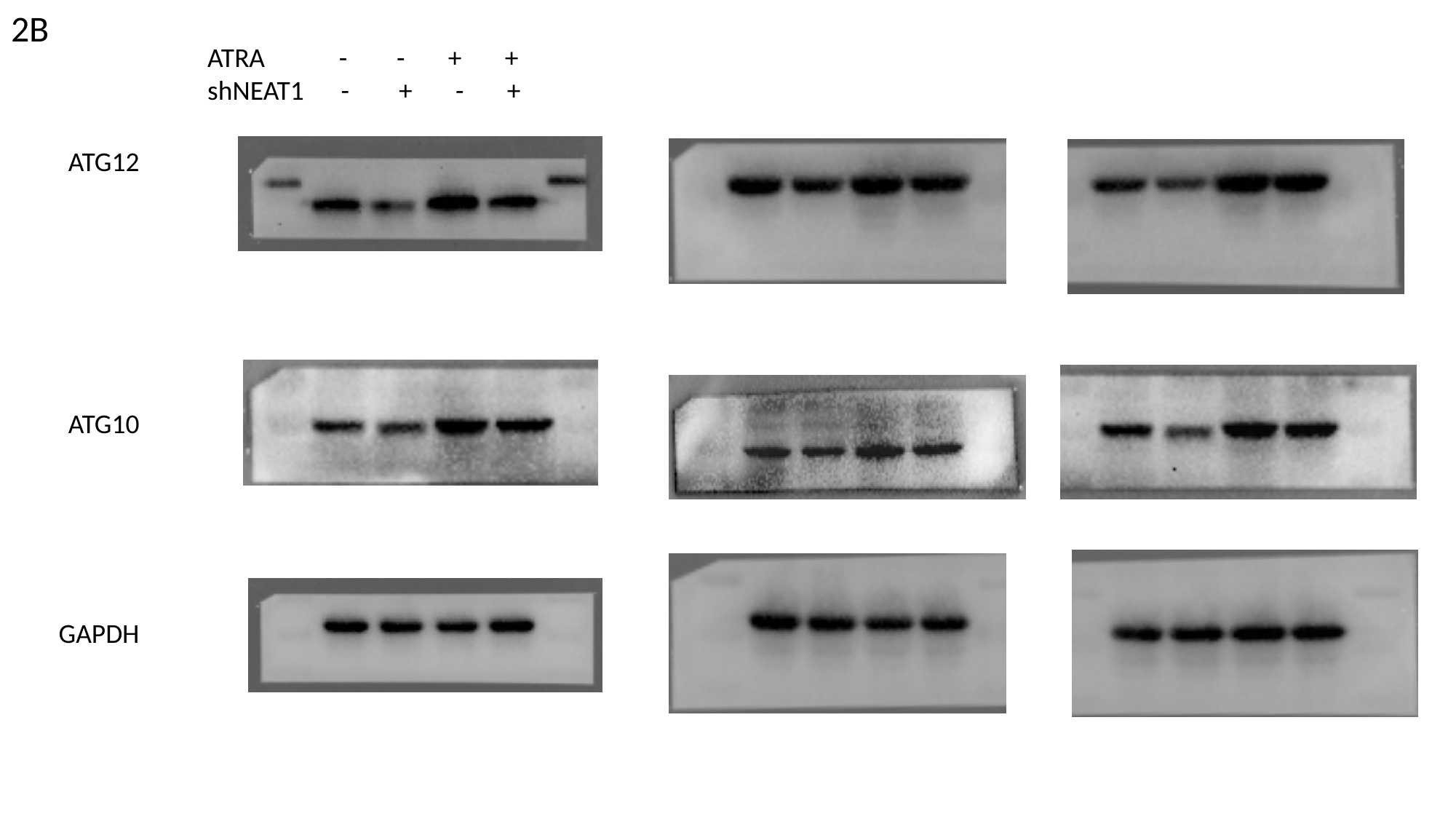

2B
ATRA - - + +
shNEAT1 - + - +
ATG12
ATG10
GAPDH

## Slide 4
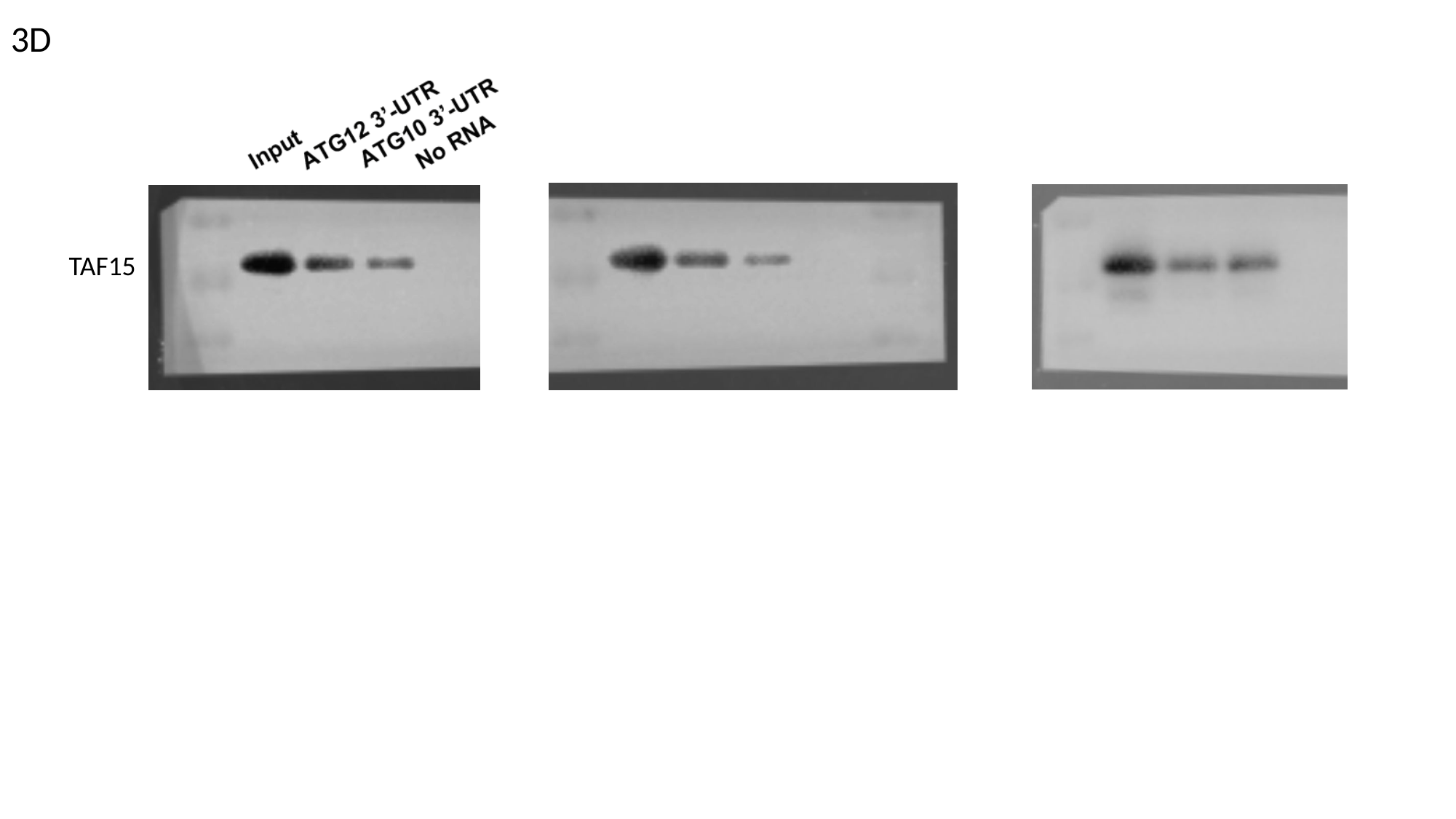

3D
TAF15

## Slide 5
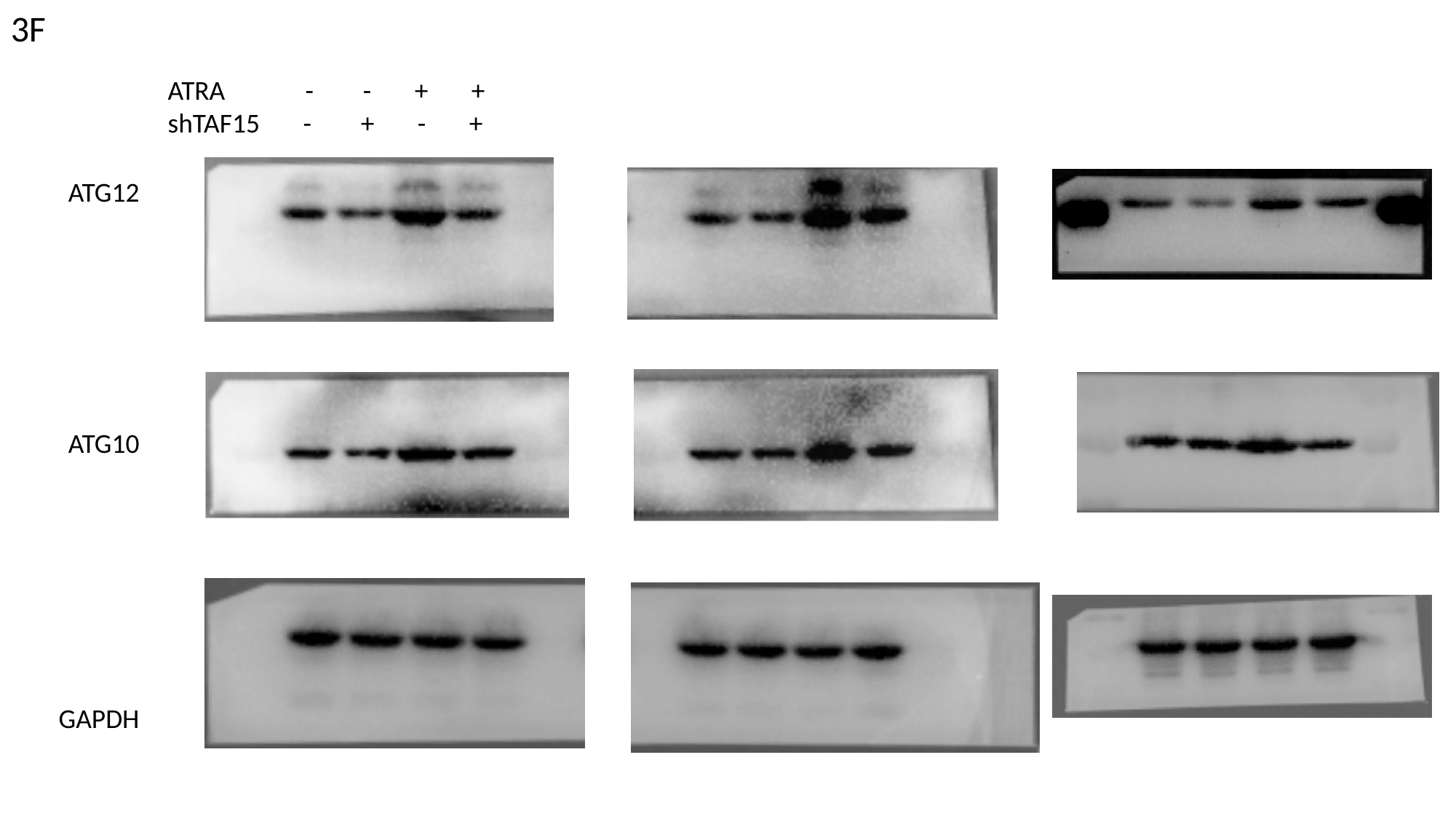

3F
ATRA - - + +
shTAF15 - + - +
ATG12
ATG10
GAPDH

## Slide 6
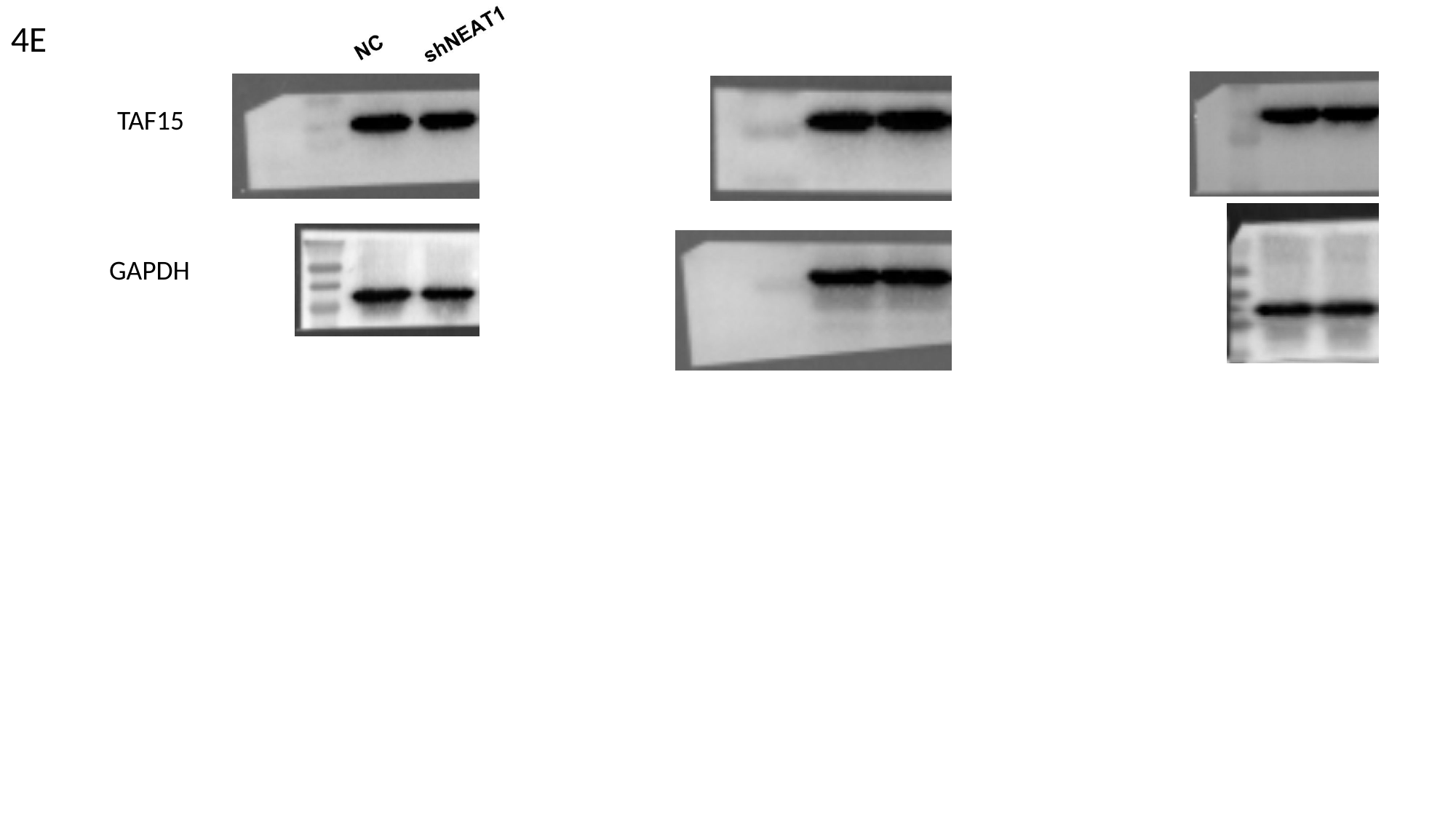

4E
TAF15
GAPDH
